# Antibiotic Resistance in Patients with Cystic Fibrosis: Past, Present, and Future

**DOI:** 10.3390/antibiotics12020217

**Published:** 2023-01-20

**Authors:** Evanthia P. Perikleous, Despoina Gkentzi, Aris Bertzouanis, Emmanouil Paraskakis, Aleksandar Sovtic, Sotirios Fouzas

**Affiliations:** 1 Medical School, Democritus University of Thrace, 68100 Alexandroupolis, Greece; 2 Department of Pediatrics, University of Patras Medical School, 26504 Patras, Greece; 3 Pediatric Respiratory Unit, University Hospital of Patras, 26504 Patras, Greece; 4 Pediatric Respiratory Unit, Department of Pediatrics, University of Crete, 71500 Heraklion, Greece; 5 School of Medicine, University of Belgrade, 11000 Belgrade, Serbia; 6 Department of Pulmonology, Mother and Child Health Institute of Serbia, 11070 Belgrade, Serbia

**Keywords:** cystic fibrosis, antibiotics, resistance

## Abstract

Patients with cystic fibrosis (CF) are repeatedly exposed to antibiotics, especially during the pulmonary exacerbations of the disease. However, the available therapeutic strategies are frequently inadequate to eradicate the involved pathogens and most importantly, facilitate the development of antimicrobial resistance (AMR). The evaluation of AMR is demanding; conventional culture-based susceptibility-testing techniques cannot account for the lung microenvironment and/or the adaptive mechanisms developed by the pathogens, such as biofilm formation. Moreover, features linked to modified pharmaco-kinetics and pulmonary parenchyma penetration make the dosing of antibiotics even more challenging. In this review, we present the existing knowledge regarding AMR in CF, we shortly review the existing therapeutic strategies, and we discuss the future directions of antimicrobial stewardship. Due to the increasing difficulty in eradicating strains that develop AMR, the appropriate management should rely on targeting the underlying resistance mechanisms; thus, the interest in novel, molecular-based diagnostic tools, such as metagenomic sequencing and next-generation transcriptomics, has increased exponentially. Moreover, since the development of new antibiotics has a slow pace, the design of effective treatment strategies to eradicate persistent infections represents an urgency that requires consorted work. In this regard, both the management and monitoring of antibiotics usage are obligatory and more relevant than ever.

## 1. Background

Cystic fibrosis (CF) is a complex, heterogeneous, multi-organ disorder caused by defects in the CF transmembrane conductance regulator (CFTR) gene. The gene encodes an ion channel that is physiologically involved in the transport of chloride and bicarbonate and is expressed on the apical epithelial cell surfaces throughout the body [1]. CFTR gene defects result in various degrees of ion channel dysfunction, thus ultimately affecting the secretory function of many organs [2]. CF is the most common autosomal recessive life-shortening condition of Caucasians; although its incidence varies significantly, it is estimated that it occurs between 1:3000 and 1:6000 live births, which equates to 1:28 and 1:40 carrier rates, respectively [3].

In the lungs, reduced chloride excretion and unrestricted sodium absorption at the airway epithelial surface result in the overproduction of thick and viscid mucus that affects airway clearance, leading to small airways plugging, irreversible structural and functional defects, and eventually, respiratory failure [4]. Repeated bacterial respiratory infections are the hallmark of the disease [4]; these lead to a domino sequence of persistent lower airway inflammation that accelerates tissue damage and accounts for the majority of the disease-related morbidity and mortality [5]. Common CF pathogens are comprised mainly of *Staphylococcus aureus* and *Pseudomonas aeruginosa*; later, as the disease progresses, many patients are infected with more uncommon and difficult-to-treat microorganisms, such as *Burkholderia cepacia*. Several complications can occur in parallel with the progression of the disease, including liver dysfunction, pancreatic exocrine insufficiency (expressed as malnutrition and failure to thrive in the pediatric population), CF-related diabetes, male infertility, nasal polyposis, intestine obstruction, bronchiectasis, and others [2,6,7].

In individuals with CF, microbial communities colonize the airways shortly after birth, while peripheral lung colonization and chronic inflammation occur later [8]. In contrast to respiratory infections in non-CF patients, these have the tendency to be persistent and involve phenotypic alterations of the infecting organisms [9]. Throughout life, patients with CF are repeatedly treated with antibiotics, especially during the exacerbations of the disease. Antibiotic administration together with novel therapeutic interventions, such as mutation-specific modulator therapies, have substantially expanded life expectancy because the latter relies mainly upon the progression of pulmonary complications [5]. However, accumulating evidence suggests the available therapeutic strategies are frequently inadequate to completely eradicate the involved pathogens [8], while standard dosing schemes may result in sub-therapeutic antibiotic levels, thus increasing the risk of treatment failure and most importantly, augmenting the appearance of antimicrobial resistance (AMR) [10]; the widespread alternating use of ‘suppressive’ and ‘curative’ antibiotic schemes increases the risk of AMR even further [11].

Notwithstanding, the evaluation of AMR is demanding; conventional laboratory antibiotic susceptibility-testing techniques rely on the interpretation of planktonic cultures, which are not entirely representative of the lung (micro) environment [12]. Indeed, the administration of an antibiotic to which a pathogen is resistant in vitro does not steadily prejudge a poor clinical result and vice versa [13]. This is an existing concern in individuals with chronic *Pseudomonas aeruginosa* and other non-fermenting Gram-negative rod (NFGR) infections [14]. Evidence has also demonstrated pathogens in CF patients commonly form biofilms, which renders them more resistant to a large spectrum of antibiotics [15]. Additionally, features linked to modified pharmacokinetics and pulmonary parenchyma penetration make the dosing of antibiotics in CF even more challenging [16].

In this review, we present the existing knowledge regarding AMR in patients with CF, we review the existing preventive strategies, and we discuss the future directions of antimicrobial stewardship considering the tremendous advancements in the domain of molecular-based diagnostic technologies.

## 2. Antibiotic Susceptibility and Biofilm Formation

**Figure 1:**
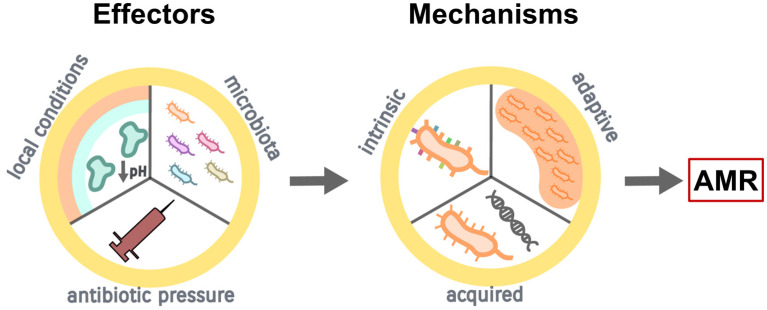
Development of antimicrobial resistance (AMR) in CF (see text for the detailed description).

CF orchestrates a diversified airway environment due to altered electrolyte levels, the presence of thick mucus, and the release of proteolytic enzymes with subsequent pH decrease; all these features are prosperous towards switching microbial growth from planktonic to biofilm type [8,17] (Figure 1). Biofilms are complex, organized, bacterial structures surrounded by an extracellular polymeric matrix composed of different macromolecules, all building a biophysical barrier that confers pathogens’ protection against the changing surrounding conditions [8,17]. Evidence suggests their formation is cued by quorum-sensing signals, i.e., extracellular chemical signals that control the expression of genes in a cell-density-dependent fashion [18]. In CF, biofilms permit the installation of a microenvironment that promotes maturation and overall shielding of pathogens against both antibiotic actions and host immune mechanisms [17,19]. Biofilm tolerance is rendered to numerous biologic processes, and it escalates as biofilm maturation progresses [19].

The presence of biofilms results in the protractedness of lung infections in patients with CF [19], ultimately contributing to the onset of pulmonary exacerbations. Current studies using culture-independent molecular techniques, such as metagenomic sequencing, have shown the complicated dynamics of airway microbiome in CF involve both the ‘traditional’, routinely identified pathogens, as well as other atypical microorganisms [20,21], as discussed below. However, antibiotics are unable to eradicate the biofilm-forming pathogens, firstly, because of their intrinsic antibiotic tolerance and secondly, due to the capacity of biofilms to favor the emergence and spreading of mutational AMR [22]. The mechanism of biofilm tolerance to antibiotics is composite and attributed to physical, physiological, and genetic factors; AMR, on the other hand, is caused by mutations following recurrent exposure to high concentrations of antibiotics [22] (Figure 1).

The formation of biofilms not only protects the bacteria against the host immune system and/or antimicrobial agents but also permits their growth and adaptation in an environment of anoxia and lack of nutrients [8]. Biofilms comprise a remarkable amount of bacterial sub-communities, which are characterized by various degrees of metabolic activity. Peripheral sub-populations demonstrate high metabolic activity, thus consuming large amounts of oxygen and nutrients; on the contrary, sub-populations located at the inner layers have lower or even zero metabolic activity, which renders them more tolerant to antimicrobial agents, thus leading to infection persistence and/or reoccurrence [23]. Higher concentrations of antibiotics or liposomal antibiotic formulations may be more effective, albeit at the price of an increased risk of both toxicity and AMR [24].

Biofilms are currently acknowledged as a fundamental driver of protracted and/or relapsing lung infections in patients with CF. Noteworthy, recently formed biofilms are much more susceptible to antibiotics than the more developed ones [25,26], thus highlighting the need for prompt and well-designed therapeutic strategies.

## 3. Microorganisms Colonizing and/or Infecting the CF Airway

The CF airway microbiota displays considerable interindividual diversity [27]. Particularly, CF patients have shown a distinguishable pattern of nasopharyngeal bacterial species, mainly attributed to the high concentration of *Staphylococcus aureus* [28,29]. This motif is established in the early years, presumably indicating the modified microenvironment of the nasal cavity that promotes the colonization by Staphylococcus while simultaneously prohibiting the adaptation and growth of commensal pathogens [30]. Consequently, the nasal cavity may reflect the initiation of S. aureus colonization of the lower respiratory tract, mainly during infancy [30]. Thus, the CF airways encompass miscellaneous nutritional and physiochemical microenvironments that guide pathogen interactions and disease pathogenesis [31].

The lower airways in patients with CF constitute a nutritionally diverse and dynamic environment that promotes long-term bacterial survival and sustains predominately polymicrobial infections, which cause significant morbidity and mortality [31]. Depending on the patient’s age, the pathogens colonizing the respiratory tract vary [17]. At birth, the respiratory tract of CF patients is supposed to be sterile; the airway microbial community develops gradually during the first two years of life and is enriched with *Streptococcus* and *Haemophilus* species [17,32]. Additionally, viral pathogens or species, such as *Mycoplasma pneumoniae* or *Chlamydophila pneumoniae*, are involved [17]. Subsequently, ordinary childhood pathogens, such as *Haemophilus influenzae* and *Streptococcus pneumoniae*, become predominant; however, they are quickly displaced initially by *Staphylococcus aureus* and later by *Pseudomonas aeruginosa* [17].

Bacterial heterogeneity is prominent in early life but decreases with increasing age and disease progression [27,33,34]. This phase is mostly associated with a rise in the prevalence and superiority of *Pseudomonas aeruginosa* [27]. *Staphylococcus aureus* and *Pseudomonas aeruginosa* usually co-exist and co-infect individuals with CF [31]. Thus, both *Staphylococcus aureus*—especially its Methicillin-resistant form *(MRSA)*—and *Pseudomonas aeruginosa* should be monitored constantly with repeated respiratory cultures [7]. Eventually, and mainly as a result of extensive antibiotic use and respiratory function decline, opportunistic pathogens, such as the *Achromobacter* species, *Stenotrophomonas maltophilia*, and other NFGR can be found [17]. Other bacterial taxa isolated in CF respiratory secretions include anaerobic species, such as *Prevotella* and *Veillonella* [31]. Furthermore, fungal and respiratory viral pathogens may be found together with bacterial microorganisms [31].

Even though eradication of early infections is attainable with appropriate antibiotic administration, protracted and chronic infections become common as patients age. Following the initial isolation of a certain pathogen, patients should be promptly and intensively treated with antibiotics to eradicate the microorganism and prevent chronic colonization and long-term adverse outcomes. The dynamic nature of the airway microenvironment and the diversity of pathogens involved, especially in chronic infections, could explain the discordance between in vitro and in vivo efficacy of antibiotics and the increased failure rate of antimicrobial treatment. The most clinically relevant pathogens are discussed below, while an overview of their AMR mechanisms is represented in Table 1; a list of empirical and most effective antibiotic modalities is presented in Table 2.

### 3.1. Pseudomonas aeruginosa

*Pseudomonas aeruginosa* is a critically important opportunistic human pathogen [35], notorious for its phenotypic flexibility and adaptability [36]. It is the most frequent cause of chronic lung infection in patients with CF, with a prevalence that increases with age: from 10 to 30% in preschoolers to more than 80% in young adults [37,38,39]. Younger patients are often colonized with multiple, wild-type, non-mucoid strains, which are gradually transformed into mucoid, biofilm-formatting pathogens [39].

As *Pseudomonas aeruginosa* has an intrinsic ability to adapt to diverse environments, it causes a variety of acute and chronic infections [35]. Chronic airway colonization is a hallmark of CF and is directly related to increased morbidity and mortality. The mean age of the initial penetration is between 6.5 and 7.1 years [40]. However, *Pseudomonas aeruginosa* isolation together with slight lung function abnormalities and computed tomography changes in infancy, are all related to a more severe disease phenotype in early childhood. Therefore, early *Pseudomonas aeruginosa* infection may be predictive of a premature impairment of lung function, thus highlighting the importance of proactive monitoring among those with early disease presentation [41]. *Pseudomonas aeruginosa* may characteristically persist in the airways of CF patients even after aggressive antibiotic treatment [42]. Early eradication with antibiotics is accomplishable, but intermittent and chronic infections appear more often with increasing age [42]. The shift from intermittent to chronic infections is mainly driven by the propensity of *Pseudomonas aeruginosa* to produce biofilms [42].

AMR in the case of *Pseudomonas aeruginosa* is not solely derived from the formation of biofilms; the bacterium is able to adapt to the surrounding environment by also presenting a variety of virulence factors (Table 1) and by the formation of multidrug-tolerant persister cells (i.e., dormant variants of cells that form randomly in microbial populations) [35,43,44]. These mechanisms often act simultaneously, resulting in AMR to almost all available antibiotics [45] (Table 1). The virulence of *Pseudomonas aeruginosa* together with its increased ability towards AMR give rise to complex and difficult-to-treat lung infections in patients with CF [35].

Generally, antibiotics are the cornerstone of CF management; the patients are subjected to repeated courses of broad-spectrum antibiotics on a chronic basis, aiming to improve their quality of life and increase life expectancy (Table 2). Antibiotics are used to eradicate *Pseudomonas aeruginosa* in the initial stages of colonization, treat pulmonary exacerbations, and control relapsing or chronic infections [42]. Early initiation of appropriate antibiotic therapy (based on the in vitro susceptibility) is substantial for treating *Pseudomonas aeruginosa* infections and protects against morbidity and severe complications [45]. However, while aggressive antibiotic treatment restricts the bacterial load, the eradication of chronic lung infection is regularly rendered ineffective [46]. The utilization of CFTR modulators and potentiators that partially restore the underlying genetic defect has shown promise even in delaying the initial colonization [47].

Currently, multidrug-resistant *Pseudomonas aeruginosa* infections represent a major healthcare concern in patients with CF, which renders the long-term management of the disease particularly challenging. In this regard, prompt and accurate knowledge of *Pseudomonas aeruginosa* AMR patterns at the individual level would be crucial.

### 3.2. Staphylococcus aureus

*Staphylococcus aureus* is among the first pathogens that colonize the respiratory tract of CF patients. Thus, it is more prevalent early in the course of the disease; in infants under the age of two years, its prevalence is higher than 50% and attains its peak value (approximately 80%) in early adolescence [48]. Staphylococcus aureus must continuously adapt to the hostile conditions of the airways to cope with host immunological responses and antibiotics and compete with other co-infecting microorganisms [48]. *Staphylococcus aureus* also acquires a biofilm mode of growth, which adds to its AMR capacity [Table 1].

In younger patients with CF, the detection of *Staphylococcus aureus* relates to significant bronchial inflammation, poorer nutritional status and overall, more severe disease [49,50]. MRSA constitutes a distinct threat to CF patients because it is characterized by more severe lung disease, increased hospitalization rates, and higher mortality [51,52]. Moreover, MRSA functions as a trigger towards failure to preserve the prior-to-infection lung function, even after administration of intravenous antibiotics for respiratory exacerbations [53]. Nevertheless, there is currently no consensus on the optimal management of both MRSA and methicillin-sensitive *Staphylococcus aureus* (MSSA) [51,52,53] (Table 2). Thus, achieving the highest level of care continues to be the target, while early eradication is desirable. Studies examining the initiation of early treatment, i.e., at the time of first MRSA-positive culture results, are imperative to guide clinical management, especially among asymptomatic individuals [53].

Although *Staphylococcus aureus* and *Pseudomonas aeruginosa* are the most predominant pathogens in CF, the latter characteristically replaces the former in the course of the disease [48,54]. However, only *Pseudomonas aeruginosa* strains that are detected in early infection clearly compete with *Staphylococcus aureus*; those isolated from chronic infection seem less antagonistic, suggesting the two pathogens are capable to interact in vivo [55,56]. Indeed, synergism interaction is presented to many CF patients with co-infections, which relate to increased rates of pulmonary exacerbations and significantly impaired pulmonary function [54].

### 3.3. Other Non-Fermenting Gram-Negative Rods

Directly in the natural course of CF, *Pseudomonas aeruginosa* and other *NFGRs* develop into cardinal species infecting and/or colonizing the lungs [17]. Thus, opportunistic pathogens, such as *Burkholderia cepacia* complex and *Achromobacter* species, *Stenotrophomonas maltophilia*, *Elizabethkingia* species, *Chryseobacterium* species, and *Alcaligenes* species, may be isolated [17,57]. Currently, the prevalence of these opportunistic pathogens is rising, predominantly due to the widespread use of antipseudomonal antibiotics [17,57].

Generally, these pathogens are broadly allocated in nature, mainly in soil and water resources [57]. However, as these species have adapted to exist in harsh environments, they are extremely difficult to be eradicated. *NFGRs* present various adaptive mechanisms resulting in significant AMR [Table 1]. Moreover, most rapid diagnostic tests are incapable to detect *NFGRs*, while institutional antibiograms scarcely include them. Consequently, both the diagnosis and treatment of these infections are tremendously challenging [57].

#### 3.3.1. *Burkholderia cepacia* Complex

The *Burkholderia cepacia* (originally called *Pseudomonas cepacia*) genus is a group of aerobic, catalase and oxidase-positive, gram-negative bacteria that includes at least 20 firmly correlated species [4,57]. All species are almost phenotypically identical and can be distinguished only by genetic or biochemical characteristics [4]. They are broadly allocated in nature, usually in soil or water sources [58], although for some of them, their reservoirs remain unknown. *Burkholderia cenocepacia, Burkholderia multivorans*, and *Burkholderia cepacia* are the most frequently isolated species [57]. Bacteria of the *Burkholderia cepacia* complex have been identified as eminent opportunistic pathogens, especially among CF patients with chronic lung infections; they can also cause nosocomial infections in immunocompromised individuals [59].

*Burkholderia cenocepacia* and *Burkholderia multivorans* represent 85% to 97% of *Burkholderia cepacia* complex infections in CF, while *Burkholderia cepacia* is more prevalent in non-CF patients [60]. These pathogens have been linked with rapid pulmonary function decline and the so-called ‘cepacia syndrome’, which can be expressed as necrotizing pneumonia and uncontrolled deterioration with septicemia and a high-mortality rate [4,60]. Individuals with *Burkholderia cenocepacia* have poorer post-lung transplant results; thus, most health institutions do not consider them as candidates for transplantation [4]. In some CF centers, single, highly transmissible clones of *Burkholderia cenocepacia* have also been reported; such strains present extremely variable virulence phenotypes, which may pose additional challenges in terms of diagnosis, treatment, and infection control [61].

*Burkholderia cepacia* complex bacteria are intrinsically resistant to many antibiotics, such as penicillins, cephalosporins, and aminoglycosides [57,60]. Despite its ability to become fatal if its management is neglected, there are currently limited clinical data to determine standard strategies for eradicating chronic *Burkholderia cepacia* complex infections in CF [60]. As a result, the treatment of these infections (Table 2) is mostly addressed by in vitro information and individual susceptibilities [57].

#### 3.3.2. *Achromobacter* species

*Achromobacter* species are motile, gram-negative, oxidase- and catalase-positive bacilli [57,62]. These opportunistic pathogens are vastly presented in aqueous environments, principally in wet soil and water resources, and in plants [62] but also in healthcare settings. Hospital outbreaks derive from contaminated solutions, such as intravenous fluids. *Achromobacter* species strains are detected in the lower and upper respiratory tract of patients with CF [62,63]. These bacteria are typically resistant to antibiotics as they comprise complex AMR mechanisms that are not fully understood to date [57].

**Table 1:** Principal opportunistic pathogens and their AMR mechanisms in CF.

Type of Resistance			
Pathogen	Intrinsic	Acquired	Adaptive
*Pseudomonas aeruginosa* [35]	Limited external membrane permeability, antibiotic-inactivating enzymes, efflux pumps	Mutational changes, overexpression, and horizontal gene transfer	Biofilm formation, persister cells
*Staphylococcus aureus* [48]	Methicillin resistance leads to resistance to all β-lactam antibiotics	Higher mutations rates and horizontal gene transfer	Biofilm formation
*Burkholderia cepacia* complex [4,57]	Antibiotic inactivation (e.g., through β-lactamases), efflux pumps, target alteration (e.g., through changed lipopolysaccharide structure)	Mutations (resistance to fluoroquinolones and trimethoprim-sulfamethoxazole)	Biofilm formation
*Achromobacter* species [57,63]	Drug inactivation (e.g., β-lactamases, and aminoglycoside-modifying enzymes), efflux pumps, changes in target production of degrading enzymes	Hypermutators in clone types (through chromosomal mutation or horizontal gene transfer)	Biofilm production

CF: cystic fibrosis, AMR: antimicrobial resistance.

*Achromobacter xylosoxidans* and *Achromobacter faecalis* are the most clinically relevant. Chronic colonization with *Achromobacter xylosoxidans* in CF patients has been associated with a significant decline in lung function and a greater risk of pulmonary exacerbations and hospitalizations [64,65,66]. In a large cohort of 1103 patients with CF in Canada, 7.3% had at least one positive culture for *Achromobacter* species [65]; individuals with chronic *Achromobacter* species infection were also at an increased risk of quick lung function deterioration, lung transplantation, and death. Currently, there are significant obstacles in the laboratory diagnosis of *Achromobacter* species, mainly due to misidentification with other Gram-negative bacilli; however, the development of novel sequencing methods, such as whole-genome sequencing (WGS), may assist towards more precise identification in clinical settings [63]. Although several clinical features and pathogenetic mechanisms remain to be clarified, Achromobacter species have been the center of increasing research interest in recent years. This is directly reflected in the number of publications regarding Achromobacter species that have tripled compared to the former decade [63].

## 4. Infection Control and Prevention

Survival in CF has improved tremendously over the last few decades with a median predicted life expectancy that currently exceeds the 40 years of age. This is the result of our enhanced comprehension of the vicious cycle of airway infection, inflammation, and tissue damage.

Awareness regarding the spread of opportunistic pathogens and the need to restrain them has determined the policy of strict segregation in healthcare settings [4]. Following patient segregation in CF medical care centers, the epidemiology has changed; the more virulent opportunistic pathogens are not as common as they were in the past, and most infections are currently due to less virulent, environmentally acquired strains [4]. Particularly in the case of the *Burkholderia cepacia* complex, segregation policies are applied to any infected member of the family [4].

Generally, to improve the quality of life and limit the days of hospitalization, there has been a shift towards ambulatory and home care rather than care in the nosocomial settings. One of the most important preventive measures is hand hygiene; additionally, the use of masks, gloves, and gowns is required for personnel in certain cases, such as colonization by multidrug-resistant bacteria or easily transmissible strains [17]. CF clinics should be organized in a manner that patients avoid unnecessary contacts to minimize the risk of transmission and acquisition of new pathogens.

There is an armamentarium of available practices and guidelines for preventing the spread of opportunistic pathogens, including standard care and contact precautions. Other important measures are antimicrobial stewardship programs aiming to promote the appropriate use of antibiotics and antibiotic de-escalation, improve patient outcomes, reduce AMR, and decrease the spread of infections caused by multi-resistant strains [67,68,69]. A detailed presentation of these practices and programs is beyond the purposes of the present review.

## 5. Future Directions

In CF, AMR continues to be a challenging health issue due to the ability of opportunistic pathogens to adapt to hostile microenvironments. Nowadays, however, the rapid progression in the domain of molecular technologies provides for the first time, the prospect for genotypic recognition of various bacterial resistance profiles [12]. Particularly, WGS data generated from next-generation sequencing (NGS) platforms provide the possibility for horizontal resistome analysis, including the detection of acquired resistance genes and relevant chromosomal mutations, thus offering greater discriminatory ability in comparison to standard molecular techniques, which rely on the pattern of nucleotide bands [70]. Furthermore, NGS has proven less time consuming and more effective in the recognition of polymicrobial communities, a task that would otherwise require repeated attempts if the standard culture-based methods were used [71].

Thus, besides applying conventional susceptibility-testing methods, screening the genome of the isolated pathogens may reveal genetic modifications that determine the phenotypic variations and the adaptation to host response that ultimately results in AMR [48]. Additionally, NGS approaches permit the elimination of culture-based bias and are extremely objective in respect to the identification of AMR factors in multimicrobial specimens [12,72]. Additionally, there are a few cardinal challenges regarding these novel sequencing processes, including the potential emergence of novel, currently unknown, resistance mechanisms, the lack of reference databases, and the fact that the recognition of certain AMR-associated genes does not automatically mean they will also be important in clinical terms [73].Genomics-based resistome analysis yields a more extensive comprehension of AMR mechanisms, which may assist in setting up auspicious novel strategies for antibiotic susceptibility testing in patients with CF [74]. By definition, resistome includes the identification of antibiotic-resistance genes and their precursors in pathogenic and non-pathogenic bacteria [70] as opposed to conventional antibiotic susceptibility testing, which targets the antibiotic susceptibility solely of pathogenic microbes. Overall, the respiratory microbiome is a dynamic and interactive network of bacteria with a set of antibiotic-resistance genes that could influence the response to antibiotics [74]. Thus, the evolution of resistome analysis might lead to individualized treatment approaches, proper prophylactic strategies, and targeted antibiotic stewardship [74].

Published data have shown the advantageous effect of resistomics in terms of predicting AMR among certain pathogens [75], providing reasonable expectations for an essential role in addressing personalized CF therapeutic interventions in the future [67]. However, the power of resistome analysis depends on which specific pathogen carries a given resistance determinant and whether in vitro susceptibilities are also important in vivo. Therefore, there are still many challenges regarding the development of resistomics-based therapeutic approaches in patients with CF.

However, even in the era of ‘resistomics’, the development of new antibiotics has been gradually reduced, and the current antimicrobial strategies are based on the combination of already existing medicines [36] (Table 2). Novel nebulized antibiotic therapies have already been introduced in clinical practice showing promising results [76]. Although inhaled antibiotics have the advantage of achieving higher concentrations in the airways while mitigating systemic toxicity, their exact place, especially with regards to the development of AMR, remains to be determined [77].

**Table 2:** Antibiotics against traditional CF pathogens.

Pathogens	Antibiotics	Type of Antibiotic	Comment
*Pseudomonas aeruginosa* [2,7,78,79]	Tobramycin * Aztreonam lysine Levofloxacin Colistimethate sodium *	Aminoglycosides Monobactams Fluoroquinolones Polymyxins	It is unclear which antibiotic option should be considered as the gold standard.
*Staphylococcus aureus* [2,13]	Vancomycin Linezolid	Glycopeptides Oxazolidinones	First-line options for CF patients with MRSA-related respiratory exacerbations.
*Burkholderia cepacia* complex ** [4,13,78]	Meropenem Trimethoprim/Sulfamethoxazol Aztreonam lysine	Carbapenems Sulfonamides/Sulfonamides Monobactams	Resistance against the majority of antibiotics.
*Achromobacter* species [13,63]	Trimethoprim/Sulfamethoxazole Ceftazidime Piperacillin Meropenem Imipenem ***	Sulfonamides/Sulfonamides Cephalosporins Extended-spectrum penicillins Carbapenems Carbapenems	Resistance against common antibiotics. They can exist for an excessive period of time in upper and lower respiratory tract.

* Available for inhalation therapy. ** Currently there have been no randomized therapeutic trials; clinicians are advised to proceed to personalized treatment. *** It is considered more active than meropenem.

Vaccines are a promising alternative to antibiotics; however, the majority are still developed by traditional methods, mainly by targeting specific antigens. Resistome analysis together with the application of new technologies, such as reverse vaccinology, could drive the design of vaccines for use in preventive and therapeutic challenges of acute and chronic infections in CF [74].

Our knowledge regarding opportunistic pathogens in CF is still deficient, impeding the emergence of effective therapeutic approaches in clinical settings. There are numerous research gaps needing additional elucidation, including the exact role of virulence factors, the mechanisms accountable for AMR, the establishment of systematic guidelines to facilitate treatment approaches, and the design of more studies in children since the extrapolation of knowledge from adult studies may not be appropriate, especially in the field of pharmacokinetics. The complexity of interspecies interactions and the dynamic nature of the respiratory environment in patients with CF represents a challenge to the development of effective preventive and therapeutic approaches [44]. Research priorities should comprise the establishment of better diagnostic tools, such as metagenomic sequencing and next-generation transcriptomics, that may directly detect AMR in the microbial communities colonizing the airways in patients with CF.

## 6. Conclusions

The presence of AMR remains a critical health issue in patients with CF. In this regard, both management and monitoring of antibiotics usage are obligatory and more relevant than ever. Due to the increasing difficulty in treating strains of *Pseudomonas aeruginosa, Staphylococcus aureus* (especially MRSA), *Burkholderia cepacia* complex, *and Achromobacter* that are commonly isolated in the respiratory tract of these patients and usually develop AMR, the appropriate management should rely on targeting the underlying resistance mechanisms; thus, the interest in novel, molecular-based diagnostic tools has increased exponentially. Moreover, since the development of new antibiotics has a slow pace, the design of effective treatment strategies to eradicate persistent infections in patients with CF represents an urgency that requires consorted work.

## Data Availability

Not applicable.
